# A Review on Recent Sensing Methods for Determining Formaldehyde in Agri-Food Chain: A Comparison with the Conventional Analytical Approaches

**DOI:** 10.3390/foods11091351

**Published:** 2022-05-06

**Authors:** Luigi Fappiano, Fabiana Carriera, Alessia Iannone, Ivan Notardonato, Pasquale Avino

**Affiliations:** Department of Agricultural, Environmental and Food Sciences (DiAAA), University of Molise, Via De Sanctis, I-86100 Campobasso, Italy; l.fappiano@studenti.unimol.it (L.F.); f.carriera@studenti.unimol.it (F.C.); a.iannone2@studenti.unimol.it (A.I.); ivan.notardonato@unimol.it (I.N.)

**Keywords:** formaldehyde, food, determination, review, sensing methods, sensors, electrochemical detection, HPLC, GC, comparison

## Abstract

Formaldehyde, the simplest molecule of the aldehyde group, is a gaseous compound at room temperature and pressure, is colorless, and has a strong, pungent odor. It is soluble in water, ethanol, and diethyl ether and is used in solution or polymerized form. Its maximum daily dosage established by the EPA is 0.2 μg g^−1^ of body weight whereas that established by the WHO is between 1.5 and 14 mg g^−1^: it is in category 1A of carcinogens by IARC. From an analytical point of view, formaldehyde is traditionally analyzed by HPLC with UV-Vis detection. Nowadays, the need to analyze this compound quickly and in situ is increasing. This work proposes a critical review of methods for analyzing formaldehyde in food using sensing methods. A search carried out on the Scopus database documented more than 50 papers published in the last 5 years. The increase in interest in the recognition of the presence of formaldehyde in food has occurred in recent years, above all due to an awareness of the damage it can cause to human health. This paper focuses on some new sensors by analyzing their performance and comparing them with various no-sensing methods but focusing on the determination of formaldehyde in food products. The sensors reported are of various types, but they all share a good LOD, good accuracy, and a reduced analysis time. Some of them are also biodegradable and others have a very low cost, many are portable and easy to use, therefore usable for the recognition of food adulterations on site.

## 1. Introduction

Nowadays, the increase in urbanization and anthropological activities has led to the release of many pollutants into the environment. The situation has meant that governments of many countries have paid attention to specific chemical elements. Among them, formaldehyde (HCHO) (FA), which is the simplest aldehyde compound, plays an important role in different fields for its toxicological implications.

FA is a colorless, highly volatile, and flammable gas at normal temperature and pressure with a strong and irritating odor. It is readily soluble in water, alcohol, and other polar solvents [1]. In the atmosphere, FA is rapidly photo-oxidized into carbon dioxide by sunlight. It reacts relatively quickly with traces of substances and pollutants in the air, so its half-life in urban air, under the influence of the sun, is short. In absence of nitrogen dioxide, the FA half-life is approximately 50 min during the day whereas in presence of nitrogen dioxide the half-life drops to 35 min [2,3].

It is produced worldwide on a large scale by catalytic, vapor-phase oxidation of methanol, and is mainly employed in several industries, such as chemicals, manufacturing, cosmetics, and textiles [4,5]. In liquid form, FA (37%) is also known as formalin, a biological preservative used for medical purposes as a embalm fluid and as a sterilizer [6,7]. Further, FA is an extremely important industrial raw material due to its chemical activity, high purity, and relative cheapness [8]; those features make it an important chemical for the global economy with an annual production of over 46 billion pounds [5].

It can be found naturally in small amounts in a wide range of raw food, including fruit and vegetables (3.3–17.3 ppm), meat (5.7–20 ppm), milk and milk products (1.0–3.3 ppm), and fish (1.0–98 ppm) [9]; in the environment, usually, it is in concentrations lower than 1 µg m^−3^ [10].

It has long been banned as a food additive and listed as a carcinogen by the International Agency for Research on Cancer and the World Health Organization. However, due to its antiseptic and preservation properties, FA is added, illegally, to food to extend the shelf life [11]. Some unscrupulous traders add or spray FA on aquatic products as a food preservative to maintain the freshness of aquatic products during transport and storage, with the potential of compromising food safety [12]. Indeed, many cases of poisoning, allergy, asthma, pulmonary damage, cancer, and death were reported as a result of formaldehyde exposure from contaminated foods, drinking water, and polluted indoor air [13]. Consequently, the development of simple and sensitive methods to monitor exposure to FA is of great interest from analytical and toxicological viewpoints [9].

## 2. Toxicology and Risk Assessment

Due to its detrimental effect on biological macromolecules, formaldehyde has been recognized since 2004 as a toxic and carcinogen substance (category 1) by international research institutions, including International Agency for Research on Cancer (IARC), United States Environmental Protection Agency (US-EPA), and the European Chemicals Agency [14]. In particular, US-EPA established that the maximum daily FA dosage is 0.2 µg g^−1^ of body weight [9,15] whereas that established by the World Health Organization (WHO) is in the range of 1.5–14 mg d^−1^ (7.75 mg d^−1^ for an average adult) [16].

Due to its cosmopolitan presence, millions of people are exposed to FA worldwide environmentally and occupationally [17], it is recognized as the third largest indoor chemical pollutant by the WHO [18]. The highest levels of this pollutant compound are found in certain occupational environments, such as industries related to manufacturing products, textile, and chemical productions, as well as medical institutions using disinfectants and embalming products [19]. Regarding the outdoor environment, FA is produced as both a primary and secondary air pollutant via atmospheric photochemistry [20] with concentrations generally below 0.001–0.002 mg m^−3^ in remote areas and urban settings, respectively [21]. Lower levels of environmental exposure come from automobile engines, household materials such as furniture made of pressed wood and carpeting, and tobacco smoke [22]. It is found that the toxicity of smoking 20 cigarettes daily is equivalent to an FA intake of 1 mg d^−1^ [6].

FA can also indirectly be produced in humans and other organisms through normal oxidative cellular and metabolic processes [17]. Possible FA exposure routes are ingestion, inhalation, absorption through the skin, and rarely blood exchange (dialysis) [23]. His endogenous concentration in the blood of human subjects is about 0.1 mM [24]. FA intake can cause chronic and acute effects that depend on many factors such as exposure time and physical fitness [25]. FA ingestion can be responsible for irritation of the eyes and the upper respiratory tract, childhood asthma, allergic skin reactions, and even nasopharyngeal cancer and potentially leukemia [4,26]. However, high and persistent levels of FA exposure can cause damage to the central nervous system, immune system disorders [27], Alzheimer’s disease [28], diabetes, chronic liver and heart disease [29], and gastrointestinal disorders [30]. Further toxicological investigations have shown also clear associations between formaldehyde exposure and tissue damage increases in cell proliferation, DNA damage, inflammation, changes in miRNA expression, and changes in gene expression signatures in direct target regions of exposure [20].

As a matter of fact, FA can stimulate the cross-linking reaction of intra- and inter-molecular including protein-protein, protein-DNA, or DNA-DNA [28]. Table 1 summarizes different health hazards caused by FA consumption discussed in this section.

Due to its acute toxicity and volatility, WHO has set a standard daily intake limit to be in the range of 0.005 × 10^−3^–0.005 mM, whilst the European Food Safety Authority (EFSA) advocates the daily oral exposure to formaldehyde from the total diet should not exceed 100 mg per day [14].

Moreover, the European Food Safety Authority (EFSA) summarized the FA level in food, for example, meat and poultry is 5.7–20 mg kg^−1^, fish is 6.4–293 mg kg^−1^, milk and milk-based products is 0.01–0.80 mg kg^−1^, sugar and sweeteners is 0.75 mg kg^−1^, fruit and vegetables are 6–35 mg kg^−1^, coffee is 3.4–16 mg kg^−1^ and alcohol beverages is 0.27–3.0 mg kg^−1^ [31].

## 3. Legislation

All over the world, various regulations have been established in concern to control the illegal use of formaldehyde in various foods and feed products [32]. In fact, the WHO, and other organizations in many countries, define the allowable regulatory values of formaldehyde concentration in the environment to avoid its influence on organisms and people [33]. The WHO has established a Tolerable Daily Intake (TDI) of 0.15 mg kg^−1^ body weight [34], the US EPA gives a reference dose (RfD) for chronic oral exposure of 0.2 mg kg^−1^ bodyweight day^−1^ [35], and most countries have set a legal limit for FA as 0.2% [15]. In China, FA is one of the prohibited preservatives mentioned in the Food Regulations, and its analysis in food is included in the food surveillance program [36]. In Europe, FA has been classified as a category 1B carcinogen by regulation (EU) no. 605/2014 [37].

In the agri-food industry, there is a legislative gap regarding the FA limit content in food because it is excluded from the list of food additives [38]. This suggests that it must be absent, but a FA limit is allowed which can migrate from the packaging to the food (15 mg kg^−1^) [39].

Therefore, to ensure the health of consumers, the FA use is still under review.

## 4. Analytical Procedure

The attention to FA detection is growing more and more in order to adopt preventive measures for the safety of public health [40]. The traditional methods such as high-performance liquid chromatography (HPLC) or gas chromatography (GC), coupled with mass spectrometer (MS) detectors, have been known to be tedious, elaborate, and high-cost analytical techniques [41]. Therefore, effective rapid and affordable methods for FA detection and quantification in food are required [42]. Nowadays, great efforts have been devoted to the development of new sensing methods, such as electrochemical, optical, and biological sensors and different kinds of probes [43]. In this review, we focus our attention on analyzing these new methods for recognizing FA and comparing their sensitivity and speed of application.

## 5. Sensing Methods for Formaldehyde

### 5.1. Scopus Data-Base and Analytical Parameter Definition

Research is currently aimed at developing increasingly low-cost systems with the aim of safeguarding the health of consumers. Given the ever-increasing attention being paid to these issues, it will be increasingly important to provide consumers with low energy consumption systems and sensors capable of detecting FA to allow for more careful monitoring. According to the WHO, for many of the diseases that arise with advancing age, we do not have efficient treatments available, nor, moreover, the specific symptoms of diseases that arise with aging could be delayed with the adoption of a healthy lifestyle. The studies for the monitoring of food conditions are placed in this context. With portable monitoring systems, it is possible to know the conditions of food in real-time, before it arrives on our tables and even, with the miniaturization of these systems, it will be possible to provide consumers when the food begins to deteriorate. In this contest, the sensing methods for determining FA play an important role in all the toxicological information above reported on this compound. The possibility to detect the FA level in any food matrix is relevant to the consumer human health protection. Following this statement, the authors performed a search on the Scopus database using different keywords, namely “formaldehyde” and “food” and “sensor” or “electrochemical detection”: 96 documents including 52 in the last 5 years that document the growing interest and importance of this type of determination. The authors focused their attention on some papers investigating the main analytical parameters and also made a comparison with traditional procedures involving chromatographic techniques for understanding the goodness and efficiency or the limits of a proposed method. The papers were divided according to the matrix investigated. Before approaching the matrices, the authors would like to resume some important definitions and concepts as well: limit of detection (LOD) and limit of quantification (LOQ), defined as the quantities of analyte that produces a signal equal to three and ten times the standard deviation of the gross blank signal, respectively [44]; relative standard deviation (RSD), calculated from results generated under repeatability (or reproducibility) conditions [45]; recovery is the yield of a preconcentration or extraction stage of an analytical process for an analyte divided by the amount of analyte in the original sample [46]. Finally, it should be remembered that reaching good recoveries suggests that the proposed system is highly accurate: in some cases, this achievement allows to suggest to the authors to support the reliability and feasibility of the developed procedure in routine analysis or real-world applications.

### 5.2. Meat

Meat is a matrix where sensing determination finds interesting applications. Table 2 resumes all the data reported in this section for the meat matrix.

First, as it can be seen, over the sensing determinations [47,48,49,50,54,55,56,57,58,59,60,61], the two tables also show the main determinations carried out by traditional techniques (HPLC, GC, spectrophotometric techniques, etc.) for a comparison of the analytical parameters and for understanding the quality level reached by the electrochemical approach [51,52,53,62,63,64]. A clear difference between the papers regarding these two matrices can be drawn immediately: the fish matrix is more studied than the other one, diverse FA sensors are investigated for different kinds of fish, whereas, chicken is the preferred meat matrix considered.

The first paper to be analyzed regards a FA rapid detection by means of a polydimethylsiloxane (PDMS) microfluidic chip [47]. Weng et al. exploited the reaction between formaldehyde and acetylacetone in presence of ammonium acetate: the compound formed (i.e., 3,5-diacetyl-1,4-dihydrolutidine) has an absorbance at 410 nm. A PDMS microfluidic chip was used for the measurements in the presence and absence of FA in the matrix. The sample to be analyzed does not undergo any strong physical-chemical treatment, basically, it is analyzed as it is, which is a strength of such measures. The authors determined different analytical parameters (limit of detection, LOD, 5.0 mg kg^−1^, recoveries between 88.6 and 110.6%, and RSD < 2.76%) but they spent time emphasizing the advantages of this method in relation to the conventional methods in terms of detection time (less than 1 min) and sample volume to be used for the analysis (1–2 µL): these allowed the authors to reduce analysis costs.

On the other hand, three recent papers deal with the FA determination in chicken samples [48,49,50]. In the first paper, Chaiendoo et al. developed a FA sensor from silver nanoclusters (AgNCs) templated by polymethacrylic acid (PMAA) [48]. The authors prepared the Tollens’ reagent ([Ag(NH_3_)_2_]^+^) from aqueous ammonia and a basic solution of Ag^+^: they used it for differentiating aldehyde from ketone functional groups due to the aldehydes properties to be easily oxidized to carboxylic acids. Simultaneously, they exploited the performance of AgNCs for increasing the detectability of Tollens’ reagent: the authors obtained the AgNCs@Tollens which reacts with FA for giving silver nanoparticles (AgNPs) increasing their size and changing the color according to the FA content. Finally, a FA chemodosimeter allows the determination at 430 nm. The authors investigated the effect both of the pH solutions (maximum absorbance at pH 4.5, it decreased to other pH values), the incubation time (mix time of AgNCs@Tollens and FA was set up at 25 min), the AgNCs concentration and of Ag^+^ concentration (0.23 mM and 0.68 mM, respectively, for the best efficiency): according to these parameters, the authors obtained a good linear response (coefficient of determination, r^2^, 0.9841) and LOD (27.99 µM), and very high recoveries in chicken (and squid) matrix (100.6–101.7%, standard deviation (sd) < 2.3), more accurate and precise than those determined by HPLC method (99.5–110.2%, sd < 6.8).

Further, the second paper, Qi et al., deals with the Tollens’ reagent: the authors made a complex with gold nanoprism and they formed a gold nanoprism/Tollens’ reagent (Au-np/TR) complex as the sensor used in headspace single-drop microextraction (HS-SDME) [49]. Basically, FA is extracted by this microextraction using Au-np/TR as a solvent droplet for reaching high enrichment. The authors focused their attention on the characterization of the complex applying techniques such as transmission electron microscopy (TEM) and energy-dispersive X-ray spectroscopy (EDX): they calculated a gold nanoparticle size of 106.5 nm as well as the presence of Ag atoms at 73.4% on the surface of the Au-np. They also studied the temperature effect on the FA reaction kinetic (best extraction temperature at 45 °C) and the extraction time (set up at 8 min). The method was evaluated in presence of different volatile organic compounds (VOCs) as interferents giving very good results (r^2^ 0.9977; relative standard deviation, RSD%, below 5%; LOD 3 nM). The authors underlined the performance of their method making a comparison with other conventional methods which displayed problems in the analysis time (up to 60 min) or in LOD values (the lowest limit reached was 100 ppb).

Finally, the third paper regards two luminescent porous networks for FA determination in aqueous media [50]. This very interesting application of the metal-organic frameworks (MOFs) allows the determination of various aldehydes (FA, butyraldehyde, valeraldehyde, propionaldehyde, 1-napthaldehyde, benzaldehyde, 4-bromobenzaldehyde, salicylaldehyde, isophthalaldehyde, etc.) in few times (1–5 min) with good LODs (0.62–1.39 μM). Basically, the authors described the performance of two solvent-dependent syntheses of Cd-based MOFs (CMERI-1 & CMERI-2); they used thermogravimetric analysis (TGA) and powder X-ray diffraction (PXRD) for characterizing their thermal and chemical stabilities. The authors also made a MOF-based hydrogel membrane, which showed the vapor-phase detection of FA. The suggestion is that these MOFs could be really useful for in situ determination of aqueous and vapor phase FA.

The other papers [51,52,53] show that conventional techniques such as paper-based analytical device (PAD), spectrophotometric technique, and solid space microextraction-gas chromatography-mass spectrometry (SPME-GC-MS) are still used for FA determination, but the levels reached by sensing methods in meat matrix are really competitive and allow to analyze the sample directly in situ. This will be a “refrain” for all the matrices but it will also be the strength of the sensing methods.

### 5.3. Fish

Really different from the meat matrix is the fish matrix in terms of papers present in literature: Table 3 shows the analytical performances of papers published in recent years (from 2015). The reason is possibly due to the fact that for a long time FA has been used as a food additive (E240) for the preservation of crustaceans (in which can be present up to values of 100 ppm) and smoked products (with higher values, up to 1000 ppm). Further, the other important reason is due to the fish degradation: after 7–10 days, or earlier if the temperature is above 0 °C, the first important alterations of the fish begin. Initially, it could be a witness to the transformation of triethylamine oxide into trimethylamine and, subsequently, into dimethylamine by bacterial and endogenous enzymes. Over time, the reaction continues, leading to the formation of monoethylamine and formaldehyde (responsible for the typical smell of spoiled fish). For these two reasons, fish is a well-studied matrix by means both of sensing methods [48,49,54,55,56,57,58,59,60,61] and no-sensing methods [51,62,63,64], especially recently favoring in situ approaches.

In this contest squid is one of the main matrices investigated: over Chaiendoo et al. [48], whose methodology was just described above, other authors have dealt with this determination in the last few years. Gu et al. [54] reported the development of an electronic nose (e-nose). The e-nose system was made by three groups of sensors, for a total of 18 sensors, showing different responses to volatile compounds in squid. The coating material of the P-type sensor was SnO_2_, whereas for T type sensor the coating materials were Pd and Pt, and for the LY-type sensor, they were Cr_2_O_3_ and Ti. The authors stated to achieve rapid (120 s) and quantitative (no analytical parameters are reported in the paper except RSDs, <0.143) FA determination whereas GC-MS analysis was used by the authors for validating the method.

Kongkaew et al. developed an electrochemical sensor made of the homogeneous distribution of palladium nanoparticle (PdNPs) on poly (acrylic acid)-functionalized graphene oxide (PAA-GO) modified on a glassy carbon electrode (GCE) (PdNPs-PAA-GO/GCE) with incorporated flow-injection amperometry (FI-Amp) [55]. TEM and Fourier transform infrared spectroscopy (FTIR) were used for characterizing the surface sensor morphology whereas cyclic voltammetry was involved in the measurements (working potential from −0.6 V to +0.7 V with scan rate 0.05 V s^−1^). The samples (i.e., squid, apple, Chinese cabbage, and cabbage) were just subjected to treatment with acetyl acetone reagent for 2 h. The authors discussed the electrochemical characterization: the formalin oxidation was obtained at −0.07 V where the sensor showed good electrocatalytic activity. Further, they addressed information about the effect both of PdNPs-PAA-GO amount (40 µg), the applied potential (−0.25 V), the flow rate (0.500 mL min^−1^), and the sample volume (250 µL). using these parameters, the authors achieved really interesting goals: an r^2^ of 0.9997 in the range 50–50,000 mmol L^−1^, LOD and LOQ of 16 mmol L^−1^ and 53 mmol L^−1^, respectively, and recoveries ranging between 94 and 104.2% with an RSD < 3.5% and no effects on the measures due to interfering ions (i.e., Cl^−^, K^+^, Na^+^, NH_4_^+^, NO_3_^−^, CO_3_^−^, SO_4_^2−^, PO_4_^3−^).

Timsorn and Wongchoosuk, on the other hand, developed a room-temperature gas sensor based on 2D hybrid pristine, NH_2_ and N_2_ functionalized multi-wall carbon nanotubes (MWCNTs)/PEDOT:PSS conductive polymer [56]. The peculiarity of this sensor is that the authors made it by inkjet printing technique: they managed to control the thickness of printed sensing films by overwriting of sensing ink on the substrate. FTIR and scanning electron microscopy (SEM) were used for characterizing the surface morphology.

A screen-printed electrode (SPE) for determining NADH and FA as well, was very recently designed by Gajjala et al. [57]. They designed a FA dehydrogenase decorated Cys-AuPd-ErGO nanocomposite with fern-like AuPd dendrites deposited on reduced graphene oxide (ErGO) on SPE. The sensor showed direct electron transfer avoiding the use of electron mediators. After sensor characterization by means of SEM, Energy-dispersive X-ray Spectroscopy (EDAX), Raman, and FT-IR spectroscopy, the authors studied the optimal conditions both for analyzing NADH and FA and for minimizing the interferences. In terms of analytical parameters, they reached a very good performance (r^2^ 0.991 in the linear range of 1–100 µM; LOD 0.3 µM; sensitivity 73 μA μM^−1^ cm^−2^). According to the interferences, they studied the selectivity related to possible agents such as acetaldehyde, uric acid, KCl, NaCl, ammonia, urea, and nitrate: different experiments were carried out and the related results showed very low interference for KCl, NaCl, ammonia, urea, and nitrate (<4.9) and acceptable one for acetaldehyde (<8.1%). Finally, the sensor was stable over two days and its activity decreased from 42% to 17% after 6–7 days which confirmed the reliability of such a developed device.

More interesting from an analytical point of view were the analytical methods developed by Rovina et al. [58]: they prepared a biodegradable hybrid polymer film where Nash colorimetric reagents were entrapped. This system was able to change color in presence of FA and the angle formed gave the FA content in the investigated matrix. The color analysis could be done by an RGB imaging system: the authors used an iPhone 10 camera equipped with a color scanning application for this kind of measurement. Different Malaysian seafoods were analyzed as real samples. After investigating the chemical-physical properties of the biodegradable film (morphology, mechanism reaction, optical analysis, structure, and mechanical properties) and optimizing the parameters (e.g., acetylacetone produced yellow color, maximum absorption at 415 nm, composition ratio, 0.054 mm thickness), the authors achieved good results in terms of coefficient of determination, 0.9918, LOD and LOQ, 5 ppm and 16.8 ppm respectively, and recoveries ranging between 98.80 and 104.65% with RSD < 1.21%.

An electrochemical sensor based on gold nanoparticles (AuNPs) and chitosan (CHIT) was reported by Noor Aini et al. [59]. They developed this biosensor and increased the electron transfer in the electrochemical cell using methylene blue as a redox indicator. Particularly, this biosensor the NADH electron from the NAD+ reduction at a potential of 0.4 V by means of differential pulse voltammetry (DPV) (optimum working conditions 0.10 V s^−1^ at pH 7.0). The core of this biosensor was the preparation of the modified electrode (FDH/AuNPS/([EMIM][OTF])/CHIT) whereas the authors assembled the electrode using a Thermo-Orion glassy carbon. Under optimum conditions, FA was detected in the range 0.01–10 ppm with LOD of 0.1 ppm and recoveries from 81.2 to 82.2% and RSD < 0.64%.

If on one side Qi et al., developed a similar sensor (AuNP) just described above [49], on the other side Yasin, et al., set up a fiber bundle-based sensor which is an optical sensor [60]: a red laser at 630 nm (obtained by He-Ne laser) with the intensity of the backscattered radiation increasing linearly with the concentration, was used for detecting FA in both snapper and gouramis fish. This system is very easy, portable, stable, and low cost, but effective for determining FA levels in the range of 3–21%.

A very recent sensor was developed by Nurley et al. [61]: they developed an optical enzymatic sensor based on MB28 copolymer membrane prepared methyl methacrylate (MMA) monomer, *n*-butyl acrylate (nBA) monomer, and benzoyl peroxide initiator. The UV-Vis spectrophotometry was involved in measuring the light absorption of the stacked membrane system for determining the FA concentration. The authors underlined the experiments performed for determining the optical absorption response in relation to the transducer membrane and optimized the immobilized enzyme, buffer pH, and buffer capacity. All the procedure was validated using the NASH standard method with the following data: r^2^ 0.9913 in the linearity range of 10^−3^–10^3^ mM, LOD 1 × 10^−6^ mM, and RSD < 7.8%, and simultaneously good statistical tests resulted comparing the method with the NASH standard method.

Finally, Table 3 also shows some analytical data from a paper reporting conventional analytical methodologies for determining FA in the fish matrix [47,51,62,63,64]: similar consideration to what above withdrawn for meat matrix can be also reported in this case. In most cases, LODs and LOQs are above those reported with sensing methods except for the determination carried out using thermal desorption and GC-MS analysis where the adsorbent is a MoO_3_/Polypyrrole (MoO_3_/PPy) which is intercalative material and possesses a large sampling capacity and good adsorption selectivity for polar compounds (very low LOD, 0.004 μg L^−1^) [64].

### 5.4. Vegetable and Fruit

Two matrices well-investigated for the FA determination are fruit and vegetables. Table 4 shows the main papers dealing with this determination and showing new analytical methods.

First, it should be noted that few papers are just discussed in the above section, the authors applied the developed procedure to these other two matrices. For instance, Kongkaew et al. [55] applied their methods developed for the squid matrix to the analysis of apple fruit samples: in this matrix, they achieved the same LOD and LOQ but reached better recoveries (100%) than the other matrix (94–104%). Tan et al. [65] reported a procedure based on a laser confocal imaging-fluorescence resonance energy transfer (TP-FRET) strategy-based TP ratiometric reversible fluorescent sensor NPXH for detecting and imaging FA. Actually, the authors developed the sensor for analyzing bisulfite, HSO_3_^−^, but after they used it for FA measures. The authors started with the idea that fluorescent sensors were a good candidate for this determination due to their excellent optical performance, high selectivity and sensitivity, non-invasiveness, and rapid response. Among different kinds of optical sensors, they chose TP radiometric fluorescent sensor because it uses low energy near-infrared light as excitation light and consequently minimum background, low light scattering, and deep penetration depth. They tested the sensor under different pH: a linear correlation in the range of 0–1 µM, a LOD of 0.00748 µM, and recoveries ranging between 98 and 100% were achieved using this approach.

An interesting paper was published by Kundu et al. in 2021 [66]: the authors studied a screen-printed electrode-based electrochemical biosensor and compared the obtained results with those obtained by means of an enzymatic optical biosensor for detecting FA in corn samples. Specifically, Kundu, et al., developed the biosensor using cyclic voltammetry technique (optimum condition 50 mV s^−1^) whereas the other used hematite nanostructure modified indium tin oxide (ITO) coated glass electrode with UV-Vis detection (200–800 nm range; absorbance at 432 nm as a result of enzyme-catalyzed reaction). After having compared the results obtained, the authors stated that the screen-printed electrodes can represent an important future perspective in this field because they can be miniaturized and easily inserted into portable instruments for detecting FA in the agri-food chain. Under optimal conditions, the authors achieved excellent performances with both the electrodes, namely sensibility (352 μA mg^−1^ L cm^−2^ using the electrochemical technique and 0.186 mg L^−1^ using the optical technique), very good LODs (0.03 mg L^−1^ and 0.02 mg L^−1^, respectively), coefficient of determination (r^2^ 0.991) in a large range (0.01–0.05 mg L^−1^) and good RSD (<2%).

Prosuwan et al. developed a novel nanocatalyst composed of Pd nanochains (PdNCs), graphene nanoflakes (GNFs), and tungsten disulfide (WS_2_) nanosheets coupled with a flow injection-based system for amperometric FA determination (PdNC-GNF/WS_2_) [67]. The authors characterized the morphology of the material by means of SEM, TEM, EDX, and FT-IR as well as optimized the parameters (i.e., the electrochemical kinetics process, the diffusion coefficient, D, the catalytic rate constant, k_cat_, the catalytic electrode stability): the results obtained analyzing FA in food samples were compared with conventional spectrophotometric analysis. Although the sensor is quite complex, its use can be very important, the authors themselves proposed this sensor for routine analysis: in fact, they specified that it can be used in Thailand, being a greater exporter of food products worldwide, both for saving the producers and buyers and for increasing human health and safety. Under the optimum conditions, the authors achieved results able to detect levels under the minimum risk quantities prescribed by various governmental agencies: linear ranges (r^2^ 0.9968) from 0.010–100 mM, sensitivity up to 220.6 μA mM^−1^ cm^2^, LOD 0.003 mM (0.10 mg L^−1^), recoveries ranging between 96 and 103% with RSD < 4%. The authors investigated the possible interferences, NH_4_^+^, Na^+^, K^+^, Cl^−^, SO_4_^2−^, CH^3^COO^−^, CO_3_^2−^, NO_3_^−^, NO_2_^−^, and urea: these interferences were evaluated not to exceed 5% of the current response change, showing that they did not interfere with the FA determination by PdNC-GNF/WS_2_ electrode.

Finally, three papers, just discussed above, report the FA determination in fruits, cereals, and vegetables by means of conventional techniques, i.e., spectrophotometric technique, SPE-GC-MS, and HPLC, respectively [52,53,63]. The first paper describes a UV-Vis procedure using Nash reagent (4-amino- 3-penten-2-one) [68] and reading the absorbance at 415 nm [52]. The authors built the linear regression equation (r^2^ 0.9209 in the range 0–10 ppm) and after analyzing different samples (bananas, carrots, tomato, radish, lemon, pineapples, mangos, pomegranates, grapes, cabbage, onion, potato, cucumber) but they did not show any other analytical parameters for evaluating the goodness of their procedure. Leong, et al., reported an SPME-GC-MS procedure: they used an SPME fiber coated with 65 μm polydimethylsiloxane/divinylbenzebe (PDMS/DVB) for adsorbing volatile compounds (over FA they also analyzed acetaldehyde) after derivatization with O-(2,3,4,5,6-pentafluoro-benzyl)-hydroxylamine hydrochloride (PFBHA) [53]. They analyzed different samples (both fruits and alcohol-free beverages) achieving very good analytical conditions: LODs ranging between 5.74 and 175 ng g^−1^ according to the matrix, linearity < 0.998 in the range 5–1000 ng g^−1^, recoveries in the range 68.37 and 128.22% and RSD < 14.53. The third paper by Wahed, et al., showed the optimization and validation of an HPLC method for determining FA in mango, rice, leafy, and fish and milk as well [63]. The authors’ intent was to harmonize the methods present in the literature regarding the HPLC-DAD (diode array detector) analysis. The determination was carried out after derivatization with 2,4-dinitrophenylhydrazine (2,4-DNPH) for forming HCHO-2,4-DNPH which is detectable at 355 nm. Particularly, r^2^ 0.99 in the range 1.0–100 m L^−1^, LOD 0.32 mg L^−1^, and LOQ 1.08 mg L^−1^ with recoveries ranging between 99.8 and 115.6% and RSD < 10.59% were the analytical parameters reached by authors under the optimal conditions: mainly, the temperature was a critical factor for the elution (at 33 °C FA co-eluted with other compounds present in the matrices, so the temperature was set up at 35–40 °C) whereas the pH solution and methanol% in the mobile phase did not affect the peak areas significantly.

### 5.5. Alcoholic and No-Alcoholic Beverages

Finally, an important matrix investigated for the FA content regards the beverage. During these last years, papers have dealt with this determination in different kinds of beverages such as alcoholic (e.g., liquor, beer, wine) and no-alcoholic (e.g., water, fruit juice. soda, coffee, soft drink) drinks: Table 5 resumes such information.

As just reported above, also in these matrices some papers were just commented on in previous sections, but the authors would like to still report the analytical parameters for a complete FA determination overview. In particular, papers dealing with the FA determination by sensing methods in water [65,67] and in fruit juice [66] were just discussed above as well as papers regarding such measurement by no-sensing methods in soft drinks (e.g., coca-cola, sprite), recreational (e.g., tea, coffee) and alcoholic (e.g., malt, wine) beverages or milk [52,53]. In all these documents very good analytical parameters were reached except for the spectrophotometric technique, used only for qualitative analysis. The use of screen-printed biosensors allowed to achieve good results also with such matrices (recoveries > 90%), LODs < 0.02 mg L^−1^, sufficient for analyzing FA according to the limits reported in the regulations.

Akshath et al. proposed a quantum dot (QD) based optical probe using non-classical cofactors for FA detection [69]. This approach was recently introduced by some authors [70,71]: they used nanoparticle probes for colorimetric/optical-based detection. Starting from this idea, Akshath et al. used an enzyme-based reaction to unlock the interaction of supramolecular nanoparticle hybrid for detecting the compound of interest: in this way, the authors solved some problems related to specificity, sensitivity, and stability in real samples, problems present in the previous papers. In particular, they used QD-gold nanoparticle (QD-GNP) and fluorescence detection: the addition of NADH enhanced the QD fluorescence allowing them to reach very low LODs (0.007 ng mL^−1^ in juice and 0.008 ng mL^−1^ in wine) and good r^2^ (0.9604 and 0.9663, respectively) and recoveries (90.9–97.2% and 91.0–98.0%, respectively). The authors developed a nano-sniffer that was stable and sensitive. Different real samples (i.e., fruit juice, and wine samples) were analyzed following the approach to sniff FA as a function of the dehydrogenase reaction.

**Table 5:** Analytical performance of sensing and no-sensing determinations applied to (alcoholic and non-alcoholic) beverage matrix. The term “N/A” means the information is absent in the paper.

Matrix	Analytical Method	LOD	LOQ	LDR	Recovery (%)	RSD (%)	Refs.
	*sensor determination*						
water	TP-FRET with fluorescent probe	0.00748 μM	N/A	0–1.0 μM	98.0–100.4	N/A	[65]
fruit juice	screen-printed biosensor	0.02 mg L^−1^	0.07 mg L^−1^	0.01–0.3 mg L^−1^	>90	<0.73	[66]
water	PdNC−GNF/WS_2_ sensor	0.10 mg L^−1^	0.33 mg L^−1^	0.01–100 mM	96–103	<1.03	[67]
juice & wine	QD-GNP ^1^	0.007 ng L^−1^	N/A	N/A	90.9–98.0	N/A	[69]
orange juice	CNT-Fe_3_O_4_ nanocomposite	0.05 mg L^−1^	N/A	0.05–0.50 mg L^−1^	>90	<1.79	[72]
liquor & beer	AuNPs/Cu,I-CD, colorimetric sensor	0.335 mg L^−1^	N/A	0.67–26.67 mg L^−1^	99.5–103.4	N/A	[73]
	*no sensing determination*						
coffee, coca-cola, malt & milk	spectrophotometric technique	N/A	N/A	0–10 ppm	N/A	N/A	[52]
tea, coffee, cola, sprite, wine, milk & milk products	SPME-GC-MS	5.74–175 ng g^−1^	N/A	50–1000 ng g^−1^	68.4~128.2	<14.53	[53]

^1^ QD-GNP: Quantum dots-Gold nanoparticle.

After the paper on screen-printed electrodes [66], Kundu et al. developed an electrochemical biosensor for determining FA adulteration in food [72]. Specifically, the group designed a nanocomposite-based biosensor for FA detection using the formaldehyde dehydrogenase (FDH) enzyme. This biosensor was made of carbon nanotubes-Fe_3_O_4_ nanocomposite (CNT-Fe_3_O_4_) by means of cyclic voltammetry. The authors exploited the interaction between FA dehydrogenase (FDH) with FA at interfaces with carboxyl-functionalized CNT, CNT–Fe_3_O_4_ nanocomposite, and co-enzyme (NADH): the result was a change in the biosensor current signal due to complex formation on the surface of the electrode. The nanocomposite was prepared for dispersion of iron oxide nanoparticles (obtained by chemical coprecipitation) in a mixture of ethanol and distilled water: the solution was kept under a magnetic stirrer for 6 h at 60 °C. Carbon nanotube and nanocomposite material were deposited onto indium tin oxide (ITO) by means of the electrophoretic deposition technique (EPD). The single materials were characterized by UV-Vis technique whereas the electrodes by FT-IR and SEM. The authors reached very good analytical parameters in the analysis of orange juices: high sensitivity (527 µA mg L^−1^ cm^−2^) in the range 0.05–0.50 mg L^−1^, LOD of 0.05 mg L^−1^, recoveries ranging between 98.2–104% with RSD < 1.15% and very long-term stability (over 70 days), very important parameter for such measurements.

Finally, a very recent paper regards the FA determination by a colorimetric sensor based on AuNPs/Cu,I-CDs composite material exhibiting high oxidase- and peroxidase-like activities [73]. These activities were used for the colorimetric detection of *tert*-butyl hydroquinone (TBHQ) whose oxidation to oxidized TBHQ can be repressed by FA. The authors prepared Cu,I-CDs nanozyme in a single step: two different sensors were developed for analyzing TBHQ and FA in liquor and beer samples whereas the compound absorption spectrum was set up at 492 nm. Under these conditions the absorbance of the red oxidized product (oxidized TBHQ) decreased with increasing FA concentration: the authors achieved a good coefficient of determination in the range 0.67–26.67 mg L^−1^ as well as satisfactory LOD of 0.335 mg L^−1^ and recoveries between 99.59 and 103.38%. The mean risk in the analysis was identified in high concentrations of interfering molecules (i.e., methanol, CH_3_CHO, phenol, MgCl_2_, CaCl_2_, KCl, NaCl) that could make minor artifacts. Further, the authors stated that in relation to parameters such as linear range and LOD their method was effective in comparison with other methods present in the literature [71,74,75,76].

### 5.6. Milk and Milk-Based Products

Another matrix largely investigated regards the milk and milk-based products: FA is added to disguise poor microbiological quality [77], and it could be used as a preservative in skimmed milk for pigs, but it is also considered an adulterant in this matrix [78]. Papers are mainly focused on FA screening. In fact, among the papers published in these recent years, two are just addressing the FA detection [79,80] whereas the other two report little analytical information [81,82]. Table 6 summarizes the main results achieved in the different papers.

Durante, et al., focused their attention on the FA real-time detection by means of applying electrical impedance measures [79]. In particular, the authors applied electrical impedance spectroscopy measurements to bovine milk samples. For this purpose, they used an LCR Meter with frequencies varying between 10 kHz and 10 MHz at 25 °C: the impedance varied as a function of FA presence in the adulterated milk as opposed to pure milk. The authors themselves showed that the presence of ions in the samples (e.g., Na^+^, K^+^, Cl^−^) did not affect the frequencies change significantly. The only parameters reported regarded the sensitivity (1% for detecting milk adulteration) and the accuracy (%) in the ranking definition (i.e., “adulterated” and “unadulterated” bovine milk). On the other hand, Saracoglu and Hayber proposed a fiber optic sensor sensitive to refractive index changes [80]. This sensor, made of plastic optical fibers, managed to analyze FA, hydrogen peroxide, and sodium carbonate at a concentration below 5%. In particular, the sensor recorded the refractive index changes in relation to the FA concentration changes in the sample. The authors focused their attention on the sensor probe development whereas no information was furnished about the analytical parameters. As stated by the authors themselves, the “main goal … is to design a sensor system being capable of detecting the possible impurities in milk for every stage of the milk processing.” The sensor is made of a multimode plastic optical fiber (POF) mounted on a sensor probe, the main part of the sensor. The light source and photodetector were a 660 nm LED and a photodiode-IC receiver, respectively. They tested 15 diverse probes with different diameters (1, 2, and 3 mm).

Xin et al. developed a fluorescent probe using a 1, 8-naphthalimides scaffold as chromophore [81]: the FA presence caused a fluorescence intensity enhancement recorded by the sensor at an excitation wavelength of 440 nm. A stable value of the fluorescence was reached within 20 min. The authors tested the FA determination in presence of other classes of compounds such as amino acids, cations, anions, reactive oxygen species, reactive nitrogen species, ketones, and aldehydes: the experiments showed that only FA provoked a clear and evident fluorescence change. The analyses were carried out at pH 7.4 in phosphate buffer saline (PBS) solution (concentration 10 mM). Under the optimal conditions, the analytical parameters achieved were the following: good linearity (r^2^ 0.998) studied in the range 0–10 µM, LOD of 1.62 × 10^−6^ M, recoveries between 49 and 112% with RSDs ranging between 14 and 0.9%.

Finally, in 2020 Veríssimo et al. published a paper regarding a sensor equipped with an optical fiber, insoluble in water, that in presence of FA gave a change in UV-Vis spectrum (329 nm) [82]. This coupling allowed the authors to achieve the advantages of electrochemical (i.e., sensor) and spectrochemical (i.e., UV-Vis) detection. A sensitive membrane, based on a polyoxometalate (POM) compound is fundamental in such sensors: in particular, the authors synthesized and characterized a [(C_4_H_9_)_4_N]_4_H[PMo_10_V_2_O_40_] material as POM for membrane prepared by mixing polyvinyl chloride (PVC) (33%), o-nitrophenyl octyl ether (NPOE) (66%) and [(C_4_H_9_)_4_N]_4_H[PMo_10_V_2_O_40_] (1%) in 1 mL of tetrahydrofuran (THF). The authors obtained very good parameters: good coefficient of determination (r^2^ 0.9994) in the range 0.6–8.5 mg L^−1^; LOD and LOQ of 0.2 mg L^−1^ and 0.6 mg L^−1^, respectively, quite similar to the conventional spectrophotometric measurements (0.2 and 0.5 mg L^−1^, respectively). Further, the authors applied their method and the conventional one to milk real samples: they found out that the measures were not statistically different.

Among the conventional methods present in literature for analyzing FA in milk samples during these last years, Wahed’s paper is noteworthy [63], just commented above (see Section 5.3 and Section 5.4): the group set up an HPLC-DAD analysis of different matrices, milk included.

### 5.7. LOD Comparison and FA Gas Sensors

Following this discussion, some analytical parameters evidenced the performances of each methodology. The authors, following a reviewer’s suggestion, would like to resume the main important parameter related to the determination, namely the LOD. Table 7 would like to resume it, independently from the analytical sensing methodology used.

Finally, the authors would like to show a particular gas sensor for analyzing FA in indoor air. Chavali et al. developed a graphene oxide (GO) based sensor for detecting FA at room temperature [83,84,85]. They largely used such nanomaterial for its physical/chemical properties [86,87,88,89,90] reaching good performances and good sensor robustness.

## 6. Conclusions

Very advanced methods are now available to improve the organoleptic characteristics and conservation of food products; for example, the introduction of the HACCP (Hazard Analysis Critical Control Point) system guarantees consumers greater safety of hygiene and healthiness of food and drinks. Unfortunately, however, sometimes even refined methods are used to commit food fraud (i.e., adulteration, alteration, counterfeiting, and sophistication), which can be defined as those modifications made to foods to derive an illicit gain. Therefore, identifying in foods the presence of substances harmful to health is certainly not easy. For all these reasons the analytical methods have really increased their performance during these two last decades. These analytical techniques are very attractive since they have several advantages: they do not require highly specialized personnel for their use and are economically more accessible than the tools most used in traditional analytical techniques. Furthermore, they also allow real-time monitoring both in the sales phases of a product and during the more delicate phases of the production process, allowing timely interventions by eliminating any delays due to the time required to carry out the analyzes in the traditional way or to receive the results of the analyzes by third-party laboratories, resulting in the optimization of the intervention by the operator. The technologies related to the sensors described here are more economically sustainable than conventional and/or traditional analytical techniques and, above all, they are able to reduce the time and cost of analyzes, while also reducing product losses due to sampling since the sample is not lost or altered during analysis.

Finally, after having highlighted advantages and, particularly, disadvantages in the text during the discussion, these authors, according to our knowledge and idea, would like to underline that some issues are really positive and can overcome the negative aspects. They believe that the possibility to get in situ analysis is really important for protecting human health without “wasting time” in collecting the food and sending it to the laboratory for analysis. Basically, the speed of response, in this case, is really important for avoiding that FA-contaminated food can reach the consumer’s table. Maybe, the operator could lose something in the precision and accuracy of the measurements but he would take a big advantage in the rapid and effective response to a policy decision. So, the authors’ idea is to improve these kinds of sensors for reaching higher accuracy and precision for detecting FA directly in situ with no chemical-physical treatments.

## Figures and Tables

**Table 1:** Hazardous effects of formaldehyde [26,27,28,29,30].

Interaction	Possible Health Hazards
Ingestion	- gastrointestinal disorders - central nervous system damage - immune system disorders - Alzheimer’s disease - diabetes - chronic liver and heart disease - DNA damage - inflammation
Inhalation	- irritation of the upper respiratory tract - childhood asthma - nasopharyngeal cancer - potentially leukemia
Absorption through the skin	- irritation of the eyes - allergic skin reactions

**Table 2:** Analytical performance of sensing and no-sensing determinations applied to meat matrix. The term “N/A” means the information is absent in the paper.

Matrix	Analytical Method	LOD ^1^	LOQ ^2^	LDR ^3^	Recovery (%)	RSD (%) ^4^	Refs.
	*sensor determination*						
pork	PDMS ^5^ microfluidic chip	5.0 mg kg^−1^	N/A ^6^	N/A	88.6–110.6	<2.76	[47]
chicken	colorimetric chemodosimeter based on AgNCs ^7^ templated by PMAA ^8^	27.99 μM	N/A	30–50 µM	99.3–110.5	<3	[48]
chicken flesh	Au-np/TR ^9^ as plasmonic sensor	3 nM	<0.05 μM	0.01–10 μM	94–107	<5	[49]
chicken	sensors by d ^10^-MOFs (CMERI-1 & CMERI-2) ^10^	0.62–1.39 μM	N/A	0.051–0.39 μM	N/A	N/A	[50]
	*no sensing determination*						
cow tripe	PAD ^11^	100 mg L^−1^	N/A	100–1000 mg L^−1^	N/A	N/A	[51]
poultry, beef, cooked, mutton	spectrophotometric technique	N/A	N/A	0–10 ppm	N/A	N/A	[52]
beef	SPME-GC-MS ^12^	25.08 ng g^−1^	N/A	100–5000 ng g^−1^	N/A	N/A	[53]

^1^ LOD: limit of detection; ^2^ LOQ: limit of quantification; ^3^ LDR: linear dynamic range; ^4^ RSD: relative standard deviation; ^5^ PDMS: polydimethylsiloxane; ^6^ N/A: not available in the paper; ^7^ AgNCs: silver nanoclusters; ^8^ PMAA: polymethacrylic acid; ^9^ Au-np/TR: gold nanoprism/Tollens’ reagent; ^10^ MOFs: metal-organic framework; ^11^ PAD: paper-based analytical device; ^12^ SPME-GC-MS: solid space microextraction-gas chromatography-mass spectrometry.

**Table 3:** Analytical performance of sensing and no-sensing determinations applied to fish matrix. The term “N/A” means the information is absent in the paper.

Matrix	Analytical Method	LOD	LOQ	LDR	Recovery (%)	RSD (%)	Refs.
	*sensor determination*						
squid	AgNCs templated by PMAA	27.99 μM	N/A	30–50 μM	100.6–101.7	<3	[48]
squid	electronic nose	N/A	N/A	ND	N/A	0.028–0.143	[54]
squid	PdNPs-PAA-GO/GCE-FI-Amp ^1^	16 mmol L^−1^	53 mmol L^−1^	50–50,000 mmol L^−1^	94–104.2	< 3.5	[55]
squid	PEDOT:PSS/MWCNTs-N_2_ sensor ^2^	1–10 ppm	N/A	10–200 ppm	N/A	N/A	[56]
fish	Cys-AuPd-ErGO on SPE ^3^	0.3 μM	N/A	1–100 μM	88–104	2.4–4.6	[57]
Seafood ^4^	biodegradable hybrid polymer film	5 ppm	16.8 ppm	0–100 ppm	98.80–104.65	0.12–1.21	[58]
seafood	electrochemical biosensors	0.1 ppm	N/A	0.01–10 ppm	81.2–82.2	0.32–0.64	[59]
octopus	Au-NP/TR as plasmonic sensor	30 nM	<50 nM	0.1–100 μM	94–98	<5	[49]
snapper, gouramis fish	fiber bundle-based sensor	N/A	N/A	3–21%	N/A	N/A	[60]
snapper fish, pomfret fish, threadfin fish	biosensor based on alcohol oxidase and pH-sensitive MB28 membrane ^5^	1 × 10^−6^ mM	N/A	10^−3^–10^3^ mM	N/A	<7.8	[61]
	*no sensing determination*						
squid	paper-based titration	100 mg mL	N/A	100–1000 mg L^−1^	N/A	N/A	[51]
fish, squid, shrimp, octopus	biodegradable colorimetric film	0.7 mg L^−1^	1.413 mg kg^−1^	0–25 mg L^−1^	N/A	0.61–3.10	[62]
fish	PDMS microfluidic chip	5.0 mg kg^−1^	N/A	5–400 mg kg	88.6–110.6	<2.76	[47]
fish	HPLC-DAD ^6^	1.75 mg L^−1^	5.83 mg L^−1^	5–100 mg L^−1^	91.2–105.3	6.72	[63]
aquatic products	MoO_3_/PPy intercalative sampling adsorbent-GC-MS	0.004 μg L^−1^	N/A	0.02–20.35 μg L^−1^	75.0–108	2.2–6.1	[64]

^1^ PdNPs-PAA-GO/GCE-FI-Amp: poly(acrylic acid)-functionalized graphene oxide modified on a glassy carbon electrode with incorporated flow-injection amperometry; ^2^ PEDOT:PSS/MWCNTs-N_2_ sensor: sensors based on 2D hybrid pristine, NH_2_ and N_2_ functionalized multi-wall carbon nanotubes conductive polymer; ^3^ SPE: screen printed electrode; ^4^ seafood: *Lutjanus erythropterus*, *Euthynnus affinis*, *Caranx indicus*, and *Penaeus monodon*; *Lutjanus malabaricus* and *Thunnus tonggol*; ^5^ MB28 methacrylic acrylic; ^6^ HPLC-DAD: High-Performance Liquid Chromatography with Diode Array Detector.

**Table 4:** Analytical performance of sensing and no-sensing determinations applied to vegetable and fruit matrix. The term “N/A” means the information is absent in the paper.

Matrix	Analytical Method	LOD	LOQ	LDR	Recovery (%)	RSD (%)	Refs.
	*sensor determination*						
apple	PdNPs-PAA-GO/GCE-FI-Amp	16 μmol L^−1^	53 μmol L^−1^	50–50,000 µM	100–101	<3.5	[55]
wolfberry	TP-FRET ^1^ with fluorescent probe	0.00748 μM	N/A	0–1.0 μM	98.0–100.4	N/A	[65]
corn	screen-printed biosensor	0.03 mg L^−1^	N/A	0.01–0.5 mg L^−1^	85.5–99.7	<1.67	[66]
corn	enzymatic optical biosensor	0.02 mg L^−1^	N/A	0.01–0.5 mg L^−1^	97–102	<2.56	[66]
tomato, cabbage, cherry	portable flow-injection amperometric sensor (Pd nanochains)	0.10 mg L^−1^	N/A	0.01–100 mM	96–103	<1.03	[67]
	*no sensing determination*						
fruit & vegetable	spectrophotometric technique	N/A	N/A	0–10 ppm	N/A	N/A	[52]
fruit & cereals	SPME-GC-MS	5.74–175 ng g^−1^	N/A	50–1000 ng g^−1^	68.4~128.2	<14.53	[53]
fruit & vegetable	HPLC-DAD	0.67 mg L^−1^	1.08 mg L^−1^	1.0–100 mg L^−1^	99.8–115.6	<10.59	[63]

^1^ TP-FRET: laser confocal imaging-fluorescence resonance energy transfer.

**Table 6:** Analytical performance of sensing and no-sensing determinations applied to milk and milk-based product matrix. The term “N/A” means the information is absent in the paper.

Matrix	Analytical Method	LOD	LOQ	LDR	Recovery (%)	RSD (%)	Refs.
	*sensor determination*						
bovine milk	electrical impedance sensor	N/A	N/A	N/A	N/A	N/A	[79]
low-fat milk	bent fiber sensor	N/A	N/A	N/A	N/A	N/A	[80]
milk	two-photon fluorescent probe	1.62 × 10^−6^ M	N/A	0–10 µM	N/A	<14	[81]
milk	optical fiber sensor with UV-Vis	0.2 mg L^−1^	0.6 mg L^−1^	0.6–8.5 mg L^−1^	N/A	N/A	[82]
	*no sensing determination*						
milk	HPLC-DAD	0.67 mg L^−1^	2.23 mg L^−1^	1.0–100 mg L^−1^	83.2–93.7	6.8–10.4	[63]

**Table 7:** Resume of the different LODs determined in the different investigated studies. The table is independent in relation to the analytical methodologies and the technologies used in the different studies.

Matrix	LOD	Refs.
pork	5.0 mg kg^−1^	[47]
chicken	27.99 μM	[48]
chicken flesh	3 nM	[49]
chicken	0.62–1.39 μM	[50]
squid	27.99 μM	[48]
squid	16 mmol L^−1^	[55]
squid	1–10 ppm	[56]
fish	0.3 μM	[57]
seafood	5 ppm	[58]
seafood	0.1 ppm	[59]
octopus	30 nM	[49]
snapper fish, pomfret fish, threadfin fish	1 × 10^−6^ mM	[61]
apple	16 μmol L^−1^	[55]
wolfberry	0.00748 μM	[65]
corn	0.03 mg L^−1^	[66]
corn	0.02 mg L^−1^	[66]
tomato, cabbage, cherry	0.10 mg L^−1^	[67]
water	0.00748 μM	[65]
fruit juice	0.02 mg L^−1^	[66]
water	0.10 mg L^−1^	[67]
juice & wine	0.007 ng L^−1^	[69]
orange juice	0.05 mg L^−1^	[72]
liquor & beer	0.335 mg L^−1^	[73]
milk	1.62 × 10^−6^ M	[81]
milk	0.27 mg L^−1^	[82]

## Data Availability

Not applicable.

## References

[1] Franz A.W., Kronemayer H., Pfeiffer D., Pilz R.D., Reuss G., Disteldorf W., Gamer A.O., Hilt A., Ley C. (2016). Formaldehyde. Ullmann’s Encyclopedia of Industrial Chemistry.

[2] World Health Organization (1989). Formaldehyde.

[3] Reingruber H., Pontel L.B. (2018). Formaldehyde metabolism and its impact on human health. Curr. Opin. Toxicol..

[4] Birkett N., Al-Zoughool M., Bird M., Baan R.A., Zielinski J., Krewski D. (2019). Overview of biological mechanisms of human carcinogens. J. Toxicol. Environ. Health Part B.

[5] Brandão P.F., Ramos R.M., Rodrigues J.A. (2018). GDME-based methodology for the determination of free formaldehyde in cosmetics and hygiene products containing formaldehyde releasers. Anal. Bioanal. Chem..

[6] Bearss J.J., Honnold S.P., Picado E.S., Davis N.M., Lackemeyer J.R. (2017). Validation and verification of steam sterilization procedures for the decontamination of biological waste in a biocontainment laboratory. Appl. Biosaf..

[7] Dorgham N.A., Dorgham D.A. (2021). Disinfectants and skin antiseptics for safe prophylaxis against covid-19, review of literature. Open Dermatol. J..

[8] Dzyadevych S.V., Arkhypova V.N., Korpan Y.I., Anna V., Soldatkin A.P., Jaffrezic-Renault N., Martelet C. (2001). Conductometric formaldehyde sensitive biosensor with specifically adapted analytical characteristics. Anal. Chim. Acta.

[9] Jinadasa B.K.K.K., Elliott C., Jayasinghe G.D.T.M. (2022). A review of the presence of formaldehyde in fish and seafood. Food Control.

[10] IARC (1995). Monographs on the Evaluation of the Carcinogenic Risk of Chemical to Humans: Formaldehyde Wood Dust and Formaldehyde.

[11] Shetty S.A., Rangiah K. (2020). Simple click chemistry-based derivatization to quantify endogenous formaldehyde in milk using ultra-high-performance liquid chromatography/tandem mass spectrometry in selected reaction monitoring mode. Rapid Commun. Mass Spectrom..

[12] Mantovani A., Aquilina G., Cubadda F., Marcon F. (2022). Risk-benefit assessment of feed additives in the one health perspective. Front. Nutr..

[13] Tang X., Bai Y., Duong A., Smith M.T., Li L., Zhang L. (2009). Formaldehyde in China: Production, consumption, exposure levels, and health effects. Environ. Int..

[14] Nishikawa A., Nagano K., Kojima H., Ogawa K. (2021). A comprehensive review of mechanistic insights into formaldehyde-induced nasal cavity carcinogenicity. Regul. Toxicol. Pharmacol..

[15] Nachaki E.O., Ndangili P.M., Naumih N.M., Masika E. (2018). Nickel-palladium-based electrochemical sensor for quantitative detection of formaldehyde. Chem. Sel..

[16] Theakston F., World Health Organization (2001). Organic Pollutants: Formaldehyde Air Quality Guidelines for Europe.

[17] Mohanty B.P., Mahanty A., Mitra T., Mohanty S., Naik A.K., Parija S.C. (2020). Proteomic and transcriptomic changes in rat liver following oral feeding of formaldehyde. Chemosphere.

[18] Burgos-Barragan G., Wit N., Meiser J., Dingler F.A., Pietzke M., Mulderrig L., Pontel L.B., Rosado I.V., Brewer T.F., Cordell R.L. (2017). Mammals divert endogenous genotoxic formaldehyde into one-carbon metabolism. Nature.

[19] Rager J.E., Smeester L., Jaspers I., Sexton K.G., Fry R.C. (2011). Epigenetic changes induced by air toxics: Formaldehyde exposure alters miRNA expression profiles in human lung cells. Environ. Health Perspect..

[20] Rager J.E., Moeller B.C., Doyle-Eisele M., Kracko D., Swenberg J.A., Fry R.C. (2013). Formaldehyde and epigenetic alterations: MicroRNA changes in the nasal epithelium of nonhuman primates. Environ. Health Perspect..

[21] IARC (2009). Monographs on the Evaluation of the Carcinogenic Risk of Chemical to Humans: Chemical Agents and Related Occupations.

[22] Lan Q., Smith M.T., Tang X., Guo W., Vermeulen R., Ji Z., Hu W., Hubbard A.E., Shen M., McHale C.M. (2015). Chromosome-wide aneuploidy study of cultured circulating myeloid progenitor cells from workers occupationally exposed to formaldehyde. Carcinogenesis.

[23] Protano C., Buomprisco G., Cammalleri V., Pocino R.N., Marotta D., Simonazzi S., Cardoni F., Petyx M., Iavicoli S., Vitali M. (2022). The carcinogenic effects of formaldehyde occupational exposure: A systematic review. Cancers.

[24] Lu K., Collins L.B., Ru H., Bermudez E., Swenberg J.A. (2010). Distribution of DNA adducts caused by inhaled formaldehyde is consistent with induction of nasal carcinoma but not leukemia. Toxicol. Sci..

[25] Bouma K., Kalsbeek-van Wijk D.K., Sijm D.T.H.M. (2022). Migration of formaldehyde from ‘biobased’ bamboo/melamine cups: A Dutch retail survey. Chemosphere.

[26] Zong J., Zhang Y.S., Zhu Y., Zhao Y., Zhang W., Zhu Y. (2018). Rapid and highly selective detection of formaldehyde in food using quartz crystal microbalance sensors based on biomimetic poly-dopamine functionalized hollow mesoporous silica spheres. Sens. Actuators B Chem..

[27] Wang X., Si Y., Wang J., Ding B., Yu Y., Al-Deyab S.S. (2012). A facile and highly sensitive colorimetric sensor for the detection of formaldehyde based on electro-spinning/netting nano-fiber/nets. Sens. Actuators B Chem..

[28] Wechakorn K., Supalang S., Suanpai S. (2020). A schiff base-based ratiometric chemosensor conjugated NBD derivative with the large stock shift for formaldehyde detection. Tetrahedron.

[29] Singha S., Jun Y.W., Bae J., Ahn K.H. (2017). Ratiometric imaging of tissue by two-photon microscopy: Observation of a high level of formaldehyde around mouse intestinal crypts. Anal. Chem..

[30] Kavian S., Azizi S.N., Ghasemi S. (2017). Novel bimetallic nanoporous Pd-Cu-SBA-16/CPE as a highly sensitive sensor for determination of formaldehyde. J. Electroanal. Chem..

[31] EFSA (2014). Scientific Opinion on the safety and efficacy of formaldehyde for all animal species based on a dossier submitted by Adiveter S.L. EFSA J..

[32] Nedellec V., Rabl A., Dab W. (2016). Public health and chronic low chlordecone exposure in Guadeloupe, Part 1: Hazards, exposure- response functions, and exposures. Environ. Health.

[33] Tsuchiya K., Furusawa H., Nomura A., Matsui H., Nihei M., Tokito S. (2019). Formaldehyde detection by a combination of formaldehyde dehydrogenase and chitosan on a sensor based on an organic field-effect transistor. Technologies.

[34] Wang I.-J., Chen C.-C., Chan C.-C., Chen P.C., Leonardi G., Wu K.-Y. (2011). A hierarchical Bayesian approach for risk assessment of melamine in infant formula based on cases of related nephrolithiasis in children. Food Addit. Contam. A.

[35] El Sayed S., Pascual L., Licchelli M., Martínez-Máñez R., Gil S., Costero A.M., Sancenón F. (2016). Chromogenic detection of aqueous formaldehyde using functionalized silica nanoparticles. ACS Appl. Mater. Inter..

[36] Wang M., Jiang S., Chen Y., Chen X., Zhao L., Zhang J., Xu J. (2012). Formaldehyde biosensor with formaldehyde dehydrogenase adsorped on carbon electrode modified with polypyrrole and carbon nanotube. Engineering.

[37] Commission Regulation (EU) (2014). Regulation amending for the purposes of introducing hazard and precautionary statements in the Croatian language and its adaptation to technical and scientific progress, Regulation (EC) No 1272/2008 of the European Parliament and of the Council on classification, labelling and packaging of substances and mixtures. Off. J. Eur. Union.

[38] Commission Regulation (EU) (2008). Regulation (EC) No 1333/2008 of the European Parliament and of the Council on food additives. Off. J. Eur. Union.

[39] Commission Regulation (EU) (2011). Commission Regulation (EU) No 10/2011 on plastic materials and articles intended to come into contact with food. Off. J. Eur. Union.

[40] Cammalleri V., Pocino R.N., Marotta D., Protano C., Sinibaldi F., Simonazzi S., Petyx M., Iavicoli S., Vitali M. (2022). Occupational scenarios and exposure assessment to formaldehyde: A systematic review. Indoor Air.

[41] Zou J., Sai T., Duan S., Winniford B., Zhang D. (2022). Automated method for short-chain aldehydes emission measurement by dynamic solid-phase microextraction on-fiber derivatization GC-MSD coupled with a flow-cell. J. Chromatogr. A.

[42] Islam S., Hasan M., Khan A.A.S., Baker M.A. (2019). A simple system to detect and measure formalin in fruit by using conductivity, pH and capacitance measurement. Carpathian J. Food Sci. Technol..

[43] Li P., Zhang D., Zhang Y., Lu W., Wang W., Chen T. (2018). Ultrafast and efficient detection of formaldehyde in aqueous solutions using chitosan-based fluorescent polymers. ACS Sens..

[44] Knoll J.E. (1985). Estimation of the limit of detection in chromatography. J. Chromatogr. Sci..

[45] Notardonato I., Gianfagna S., Castoria R., Ianiri G., De Curtis F., Russo M.V., Avino P. (2021). Critical review of the analytical methods for determining the mycotoxin patulin in food matrices. Rev. Anal. Chem..

[46] Burns D.T., Danzer K., Townshend A. (2002). Use of the terms “recovery” and “apparent recovery” in analytical procedures. Pure Appl. Chem..

[47] Weng X., Chon C.H., Jiang H., Li D. (2009). Rapid detection of formaldehyde concentration in food on a polydimethylsiloxane (PDMS) microfluidic chip. Food Chem..

[48] Chaiendoo K., Sooksin S., Kulchat S., Promarak V., Tuntulani T., Ngeontae W. (2018). A new formaldehyde sensor from silver nanoclusters modified Tollens’ reagent. Food Chem..

[49] Qi T., Xu M., Yao Y., Chen W., Xu M., Tang S., Shen W., Koing D., Cai X., Shi H. (2020). Gold nanoprism/Tollens’ reagent complex as plasmonic sensor in headspace single-drop microextraction for colorimetric detection of formaldehyde in food samples using smartphone readout. Talanta.

[50] Bej S., Mandal S., Mondal A., Pal T.K., Banerjee P. (2021). Solvothermal synthesis of high-performance d^10^-MOFs with hydrogel membranes @ “Turn-On” monitoring of formaldehyde in solution and vapor phase. ACS Appl. Mater. Interfaces.

[51] Taprab N., Sameenoi Y. (2019). Rapid screening of formaldehyde in food using paper-based titration. Anal. Chim. Acta.

[52] Nowshad F., Islam M.d.N., Khan M.S. (2018). Concentration and formation behavior of naturally occurring formaldehyde in foods. Agric. Food Secur..

[53] Jeong H.S., Chung H., Song S.H., Kim C.I., Lee J.G., Kim Y.S. (2015). Validation and determination of the contents of acetaldehyde and formaldehyde in foods. Toxicol. Res..

[54] Gu D.-G., Liu W., Yan Y., We W., Gan J.H., Lu Y., Jang Z.-L., Wang X.-C., Xu C.H. (2019). A novel method for rapid quantitative evaluating formaldehyde in squid T based on electronic nose. LWT.

[55] Kongkaew S., Kanatharana P., Thavarungkul P., Limbut W. (2017). A preparation of homogeneous distribution of palladium nanoparticle on poly (acrylic acid) functionalized graphene oxide modified electrode for formalin oxidation. Electrochim. Acta.

[56] Timsorn K., Wongchoosuk C. (2019). Inkjet printing of room-temperature gas sensors for identification of formalin contamination in squids. J. Mater. Sci. Mater. Electron..

[57] DeGajjala R.K.R., Gade P.S., Bhatt P., Vishwakarma N., Singh S. (2022). Enzyme decorated dendritic bimetallic nanocomposite biosensor for detection of HCHO. Talanta.

[58] Rovina K., Vonnie J.M., Shaeera S.N., Yi S.X., Halid N.F.A. (2020). Development of biodegradable hybrid polymer film for detec-tion of formaldehyde in seafood products. Sens. Bio.-Sens. Res..

[59] Noor Aini B., Siddiquee S., Ampon K. (2016). Development of formaldehyde biosensor for determination of formalin in fish samples; Malabar Red Snapper (*Lutjanus malabaricus*) and Longtail Tuna (*Thunnus tonggol*). Biosensors.

[60] Yasin M., Irawat N., Zaidan A.H., Mukti A.T., Soegianto A., Rosalia D.K.P., Wardanid R.A., Khasanah M., Kbashi H.J., Perego A.M. (2019). Fiber bundle sensor for detection of formaldehyde concentration in fish. Opt. Fiber Technol..

[61] Ahmad M., Heng L.Y., Tan L.L. (2022). Optical enzymatic formaldehyde biosensor based on alcohol oxidase and pH-sensitive methacrylic-acrylic optode membrane. Spectrochim. Acta A.

[62] Wongniramaikul W., Limsakul W., Choodum A. (2018). A biodegradable colorimetric film for rapid low-cost field determination of formaldehyde contamination by digital image colorimetry. Food Chem..

[63] Wahed P., Razzaq M.d.A., Dharmapuri S., Corrales M. (2016). Determination of formaldehyde in food and feed by an in-house validated HPLC method. Food Chem..

[64] Ma Y., Zhao C., Zhan Y., Li J., Zhang Z., Li G. (2015). Separation and analysis of trace volatile formaldehyde in aquatic products by a MoO_3_/polypyrrole intercalative sampling adsorbent with thermal desorption gas chromatography and mass spectrometry. J. Sep. Sci..

[65] Tan L., Ding H., Chanmungkalakul S., Peng L., Yuan G., Yang Q., Liu X.L., Zhou L. (2021). A smart TP-FRET-based ratiometric fluorescent sensor for bisulfite/formaldehyde detection and its imaging application. Sens. Actuators B Chem..

[66] Kundu M., Krishnan P., Gajjala S. (2021). Comparative studies of screen-printed electrode based electrochemical biosensor with the optical biosensor for formaldehyde detection in corn. Food Bioprocess Technol..

[67] Promsuwan K., Saichanapan J., Soleh A., Saisahas K., Kanatharana P., Thavarungkul P., Tayayuth K., Guo C.X., Li C.M., Limbt W. (2021). Portable flow injection amperometric sensor consisting of Pd nanochains, graphene nanoflakes, and WS_2_ nanosheets for formaldehyde detection. ACS Appl. Nano Mater..

[68] Compton B.J., Purdy W.C. (1980). The mechanism of the reaction of the Nash and the Sawicki aldehyde reagent. Can. J. Chem..

[69] Akshatha U.S., Bhatt P. (2018). Supramolecular nano-sniffers for ultrasensitive detection of formaldehyde. Biosens. Bioelectron..

[70] Wang Y., Deng X., Liu J., Tang H., Jiang J. (2013). Surface enhanced Raman scattering based sensitive detection of histone demethylase activity using a formaldehyde-selective reactive probe. Chem. Commun..

[71] Zeng J.-B., Fan S.-G., Zhao C.-Y., Wang Q.-R., Zhou T.-Y., Chen X., Yan Z.-F., Li Y.-P., Xing W., Wang X.-D. (2014). A colorimetric agarose gel for formaldehyde measurement based on nanotechnology involving Tollens reaction. Chem. Commun..

[72] Kundu M., Bhardwaj H., Pandey M.K., Krishnam P., Kotnala R.K., Sumana G. (2019). Development of electrochemical biosensor based on CNT–Fe_3_O_4_ nanocomposite to determine formaldehyde adulteration in orange juice. J. Food Sci. Technol..

[73] Li Q., Yang D., Tammina S.K., Yang Y. (2022). Construction of AuNPs/Cu,I-CD-based colorimetric sensor: Catalytic oxidation of TBHQ and the catalytic inhibition of HCHO. Food Chem..

[74] Ge W., Chang Y., Natarajan V., Feng Z., Zhan J., Ma X. (2018). In2O3-SnO2 hybrid porous nanostructures delivering enhanced formaldehyde sensing performance. J. Alloys Compd..

[75] Ma W., Row K.H. (2018). Solid-phase extraction of chlorophenols in seawater using a magnetic ionic liquid molecularly imprinted polymer with incorporated silicon dioxide as a sorbent. J. Chromatogr. A.

[76] Im D., Kim D., Jeong D., Park W.I., Chun M., Park J.-S., Kim H., Jung H. (2020). Improved formaldehyde gas sensing properties of well-controlled Au nanoparticle-decorated In2O3 nanofibers integrated on low power MEMS platform. J. Mater. Sci. Technol..

[77] Hansen P.W., Holroyd S.E. (2019). Development and application of Fourier transform infrared spectroscopy for detection of milk adulteration in practice. Int. J. Dairy Tech..

[78] Gondim C.D., Junqueira R.G., de Souza S.V.C., Ruisanchez I., Callao M.P. (2017). Detection of several common adulterants in raw milk by MID-infrared spectroscopy and one-class and multi-class multivariate strategies. Food Chem..

[79] Durante G., Becari W., Lima F.A.S., Peres H.E.M. (2016). Electrical impedance sensor for real-time detection of bovine milk adulteration. IEEE Sens. J..

[80] Saracoglu O.G., Hayber S.E. (2016). Bent fiber sensor for preservative detection in milk. Sensors.

[81] Xin F., Tian Y., Jing J., Zhang X. (2019). A two-photon fluorescent probe for imaging of endogenous formaldehyde in HeLa cells and quantitative detection of basal formaldehyde in milk samples. Anal. Methods.

[82] Verissimo M.I., Gamelas J.A., Fernandes A.J., Evtuguin D.V., Gomes M.T.S. (2020). A new formaldehyde optical sensor: Detecting milk adulteration. Food Chem..

[83] Yu M.-R., Wu R.-J., Suyambrakasam G., Joly J., Chavali M. (2012). Evaluation of graphene oxide material as formaldehyde gas sensor. Adv. Sci. Lett..

[84] Wu R.-J., Liu Y.-S., Lai H.-F., Wang J.H., Chavali M. (2014). Promotion effect of Pd on TiO_2_ for visible light photocatalytic degradation of gaseous formaldehyde. J. Nanosci. Nanotechnol..

[85] Chavali M., Srinivasan K., Wu R.J. (2018). Sensing formaldehyde using graphene oxide as sensing material. Res. Dev. Mater. Sci..

[86] Mattevi C., Eda G., Agnoli S., Miller S., Mkhoyan K.A., Celik O., Mastrogiovanni M., Granozzi G., Garfunkel E., Chhowalla M. (2009). Evolution of electrical, chemical, and structural properties of transparent and conducting chemically derived graphene thin films. Adv. Funct. Mater..

[87] Bourlinos A.B., Gournis D., Petridis D., Szabo T., Szeri A., Dékány I. (2003). Graphite oxide: Chemical reduction to graphite and surface modification with primary aliphatic amines and amino acids. Langmuir.

[88] Li J., Vaisman L., Marom G., Kim J.K. (2007). Br treated graphite nanoplatelets for improved electrical conductivity of polymer composites. Carbon.

[89] Seredych M., Pietrzak R., Bandosz T.J. (2007). Role of graphite oxide (GO) and polyaniline (PANI) in NO_2_ reduction on GO-PANI composites. Ind. Eng. Chem. Res..

[90] Joung D., Chunder A., Zhai L., Khondaker S.I. (2010). High yield fabrication of chemically reduced graphene oxide field effect transistors by dielectrophoresis. Nanotechnology.

